# Mixed Reality for a collective and adaptive mental health metaverse

**DOI:** 10.3389/fpsyt.2023.1272783

**Published:** 2024-01-05

**Authors:** Samuel Navas-Medrano, Jose L. Soler-Dominguez, Patricia Pons

**Affiliations:** Instituto Tecnológico de Informática, Valencia, Spain

**Keywords:** metaverse, extended reality, mental health, Mixed Reality, adaptive interactions, collaborative experience

## Abstract

This research paper explores the significant transformative potential of Mixed Reality (MR) technology as enabler of the metaverse, specifically aimed at enhancing mental health therapies. The emerging world of the metaverse, a multiuser, adaptive, three-dimensional digital space, paired with the interactive and immersive benefits of MR technology, promises a paradigm shift in how mental health support is delivered. Unlike traditional platforms, MR allows for therapy within the comfort of the user's familiar surroundings, while incorporating the benefits of social collaboration and interactions. The metaverse environment fosters heightened personalization and deeper user engagement, thereby offering a more tailored approach to computerized therapy. Beyond its immersive capabilities, MR offers potential for real-time, smart adaptations to the users' psycho-physiological state, targeting unique patients' needs on a diverse spectrum of therapeutic techniques, thus broadening the scope of mental health support. Furthermore, it opens avenues for continuous emotional support in everyday life situations. This research discusses the benefits and potentials of integrating MR within a mental health metaverse, highlighting how this innovative approach could significantly complement traditional therapeutic methods, fostering improved treatment efficacy, focusing on social and collective experiences, and increasing patient engagement.

## 1 Introduction

In recent decades, mental health concerns have garnered significant attention as they emerge as one of the most pressing global public health issues. The prevalence of mental health disorders has been on a steady rise, impacting individuals from all walks of life and transcending geographical boundaries (1). Historically, mental health treatment predominantly focused on individual therapy, which currently accounts for 95% of private practitioners' sessions (2). However, in the wake of increased recognition of the social dimensions of mental health and the powerful impact of community support, the paradigm of mental health treatment has evolved over time. Group therapy and support groups have established as complementary interventions to individual therapy, offering distinct benefits. For example, fostering a sense of belonging and connection (3). In addition, support groups help reduce feelings of isolation and stigmatization, offering participants an environment where they can be understood and validated by their peers navigating similar struggles (4). These groups also offer a unique platform for participants to witness the growth and progress of others, providing a sense of hope and inspiration (5).

The adoption of group therapy for addressing mental health challenges is not without barriers (6–8). Geographical limitations, such as inaccessibility to mental health resources in rural areas, hinder participation. Stigma surrounding mental health may deter individuals from joining group sessions (9). Pandemics and crisis situations pose unique obstacles, necessitating virtual platforms that may be inaccessible to some due to technological limitations. Interpersonal challenges within groups can affect therapeutic efficacy. In certain situations, such as severe mental health conditions, cognitive impairments, or with individuals exhibiting potentially harmful behavior, group therapies may not be immediately feasible or suitable. Cultural and language barriers can also impact communication and trust (10). Addressing these challenges is essential to create inclusive and effective therapeutic environments, fostering collective healing and resilience in the face of mental health struggles.

**Figure 1:**
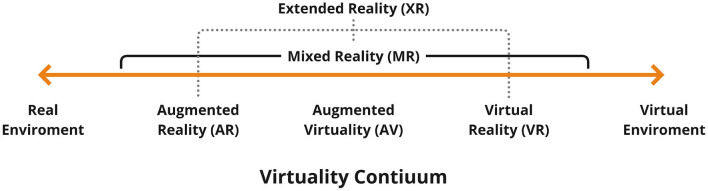
Extended reality continuum [adapted by (12) from (11)].

Emerging technologies, such as Extended Reality (XR), offer promising alternatives to overcome such barriers. XR refers to a spectrum of technologies that blend the physical and digital realms (11) (see Figure 1). At one end of this spectrum, Virtual Reality (VR) provides users with complete immersion into computer-generated environments, typically achieved through head-mounted displays (HMDs) that block out the physical world. At the opposite end, Augmented Reality (AR), overlays digital elements onto the user's physical environment, but with limited interactive capabilities. Mixed Reality (MR) bridges the gap between VR and AR, allowing users to interact with both virtual and physical elements in real-time and within the same spatial context.

In this regard, immersive environments are capable of eliciting emotional responses in the person using them (13), making XR-based interventions very useful tools to address different aspects of mental health. Transversal to mental health, emotional regulation (ER) studies how individuals influence which emotions they have, when they have them, and how they experience and express them (14). There is consistent evidence showing the presence of ER difficulties in a wide range of mental disorders, suggesting that emotion dysregulation is an important factor to target in clinical interventions (15). In addition, the social dimension is also very relevant for ER: social interactions and interpersonal relations might elicit different emotional responses or even be used after emotional experiences to help individual's regulation (16). Hence, XR arises as a powerful means to practice emotional regulation strategies in a social and multicultural environment, opening a wide range of possibilities to create immersive and collective therapeutic activities. This manuscript will review how XR technologies can help support emotional regulation practices (Section 2), discuss how MR could provide the basis for a mental health metaverse that addresses ER strategies, its the benefits and potentials (Section 3), and concludes by highlighting how current and future research could help build such an accessible and inclusive mental health metaverse.

## 2 Literature review

### 2.1 Supporting mental health in XR through emotional regulation strategies

XR technologies have been widely used in mental health for the past two decades, showing promising potential in helping individuals learn and practice emotional regulation skills. VR and AR are especially interesting as a means to conduct Exposure Therapy (ET). They allow replicating scenarios in a simulated and controlled manner for progressive exposure, making it ideal for exposing patients to a scenario that elicits a specific emotion in them. VR also helps patients who might experience difficulties engaging in visualization-based practices often required when using ET techniques. Related work has explored the use of VR for ET in the treatment of various phobias (17–20), post-traumatic stress disorder (21), or to overcome social phobia (22). VR is also a helpful resource for treating eating disorders, reducing food-related craving and anxiety by gradual exposure to virtual representations of food (23), or using digital avatars to diminish negative body-related emotions in Mirror exposure therapy (MET) (24).

Attentional strategies such as mindfulness, breathing exercises or relaxation techniques, can also be supported by XR experiences. ZenG (25) proposes a MR application for kinesthetic meditation, i.e., centered on activities such as walking, gardening, etc. Based on the cognitive state captured by an EEG device, the color of the environment will change to show how the user is performing. Instead, Amores et al. (26) focused on internal reflection, developing a VR environment that procedurally generates 3D creatures, and changes the lighting of the environment to reflect users' internal state based on EEG, EDA and HR.

Breathing exercises have shown to decrease stress and improve feelings of relaxation, and their practice can be supported by digital games aimed at improving users' wellbeing. For example, Life Tree (27) is a VR experience that proposes three different scenarios to practice breathing exercises by using breathing biofeedback. Another VR game is DEEP (28), designed for children with anxiety, who are encouraged to explore an underwater fantasy world to practice diaphragmatic breathing.

Several works have focused on serious games and game mechanics to offer alternative ways of approaching adolescents mental health. For example, InMind is a VR game explicitly designed to intervene in people's beliefs about the malleability of emotions (29). Thanks to game-based mechanics, VR therapeutic experiences can increase adolescents engagement, attendance and adherence to treatment in comparison with traditional interventions like role-plays (30).

Various XR therapeutic interventions aimed for individuals reported that participants around the patient using the device also intervened in the therapeutic activity (31, 32), either providing advice or asking questions to the current participant. Sharing the same physical space in XR interventions can increase socialization and interactions between patients and therapists. However, remote therapeutic interventions could also benefit from group sessions: Dilgul et al. (33) evaluated a VR social experience to deliver cognitive behavioral group therapy (CBGT) for patients with depression. The VR scenario allowed patients to interact with each others remotely via avatars, and this anonymity increased participation and patients' willingness to talk more honestly.

There is a growing interest in recent years in applying XR technologies for ER settings (34). However, the majority of related works are based on immersive ET, focusing on reproducing real world settings within controllable and customizable virtual scenarios. This leaves unexplored plenty of other potential therapeutic approaches that are based on more creative contexts, showing that the whole potential of XR technologies is far from being fully achieved. Especially, there is a lack of shared, social experiences and group activities, and the use of game mechanics as a way to motivate participants and encourage adherence to the therapy should be further explored. In addition, each XR scenario is usually designed and implemented to treat specific and precise mental health issues, and might not be applicable to other diagnoses or patients. Different technological solutions might present different architectures and even the supported devices which might not be compatible. This is an inefficient process for therapists and professionals, who do not have time to incorporate several XR solutions to their practice. Therefore, there is need for a cohesive and integrative platform that supports a variety of devices, therapeutic activities and customization options: the mental health metaverse.

### 2.2 A brief introduction to the Metaverse

The term “Metaverse” has become a focal point of curiosity, expectations, fears, and uncertainties in the modern era. As part of the postmodern quest to coin new terms that mark uncharted territories, the concept of the Metaverse represents a new dimension of the internet, promising to revolutionize our lives permanently. Metaverse encompasses a broader concept that includes virtual worlds, extended reality (XR) experiences, and other digital spaces where users can interact with each other and digital content in real-time. It refers to a shared, interconnected, and persistent virtual space that goes beyond individual applications or games. Users can create avatars, socialize, trade, conduct business, and engage in various activities within these virtual environments.

The Metaverse concept can be materialized accross different technologies, being those under the XR umbrella the most appropriate, mostly by their immersive capabilities. Immersion (35), represents the capacity of a technology to deliver highly engaging and interactive experiences, fully transporting users into virtual, augmented, or mixed environments. XR technologies strive to blur the boundaries between the physical and digital worlds, creating a strong sense of presence and involvement within the digital environment. From immersion, it could be inferred the feeling of presence. The sense of presence (36), traditionally associated to the feeling of “being there (in the digital world)" is strongly related to non-mediated technology-based experiences and suspension of disbelief. The concept of presence is multidimensional, attending to the wide XR spectrum consists of three major dimensions. The first one is telepresence, which is characterized by the extent to which a user experiences a sense of “reality" within the virtual environment as opposed to the physical one (37). This dimension of presence is associated to Virtual Reality. The second dimension refers to local presence, indicating the extent to which a user perceives augmented reality (AR) objects as truly existing in their immediate physical surroundings (38). This dimension is closely linked to Augmented Reality and Mixed Reality. The third dimension, co-presence, is limited to multi-user immersive experiences and refers to the psychological connection between participants and how real those social interactions are perceived to be (39).

The Metaverse builds upon the capabilities of these XR technologies, weaving them together to form a collective virtual universe that enables social interactions, commerce, entertainment, and educational experiences, fostering the three dimensions of presence previously defined. While VR focuses on transporting users to entirely digital environments (telepresence), AR (local presence) enhances the real world with digital overlays. MR (local presence) augments the feeling of presence since holograms are able to interact with the real world. All three approaches are able to implement co-presence scenarios, with higher or lower level of presence, since they all can be set up as multi-user environments.

## 3 The mental health metaverse

### 3.1 The role of Mixed Reality in a social metaverse

As can be observed in the literature of XR for mental health, VR and AR are the most common technologies being used. However, the devices used for the deployment of this type of applications are often not very accessible and inclusive. For example, VR implies that the person must wear glasses that visually isolate them from the real location where they are, so that the relationship between what the eyes perceive in the virtual world and the physical movements perceived through the vestibular system of the ear (responsible for spatial orientation and balance) is lost. Hence, VR sometimes leads to severe Visual Induced Motion Sickness (VIMS) and accessibility problems (40), especially in people with high levels of stress (41). In the case of AR, its consumption is often linked to devices such as mobiles or tablets, thus sacrificing almost all of the immersion, but, as a trade-off, they have almost no VIMS problems (42, 43).

In contrast, in the field of mental health, there are hardly any interventions that make use of MR. However, MR constitutes a very interesting intermediate point between VR and AR: it allows a relevant degree of immersion, fostering local and co-presence, while avoiding VIMS problems, and in turn offers a wide variety of possibilities for natural interaction and the combination of the physical and digital worlds (44). This combination of both worlds allows to create rich scenarios, and paves the way toward more social and co-located experiences, which could be applied both in remote or in-person group therapies. Remote collaborative activities could be mediated by the use of avatars, that will be displayed by the HMD in the real environment of each participant. In-person group therapies will involve participants in the same physical spaces, some or all of them wearing MR headsets in which the same digital scenario is shared. In addition, MR facilitates that users perform their therapeutic activities in any space: MR applications can be designed to recognize surfaces and obstacles thanks to the depth cameras of MR devices, hence the digital content can adequate itself to the identified location and elements. It also allows therapists to have an intermediate step between practicing therapeutic activities in the clinic, and performing them in daily life, by first practicing using MR support in one's own real environment.

### 3.2 Adaptive and customizable experiences

Given the great variability of possible situations, the personal evolution and progression of each person, as well as individual preferences and responses, it is complex to configure this type of environments manually. Some works propose variants of each exercise with different difficulties (32). Other works offer tools to support the therapist's decision. For example, Heyse et al. (45) have developed a prototype adaptive algorithm for VR exposure therapy that automatically offers the therapist four different configurations to choose from, based on the patient's data, as well as allowing the modification of certain parameters such as blurring or flickering lights. This makes it easy to configure and customize the scenario without the need for technical expertise. However, these approaches are costly because they involve different developments and are not suitable for all needs.

Therefore, an essential aspect in this type of environments is that the XR experience intelligently adapts its content and goals to the user's need, instead of requiring therapists to manually set up each possible configuration. In this way, instead of preparing multiple options and outcomes for the same scenario, designers and developers could focus their efforts in the implementation of brand new therapeutic activities, that will adapt autonomously based on the necessary parameters defined by the professionals. In this regard, adaptive XR scenarios can leverage the potential of users' biofeedback to improve their response to stimuli and to adapt the environment to the defined parameters in an intelligent and automatic way. This biofeedback has to be presented in a way that does not evoke categorizations of behavior as right or wrong, i.e., the user does not feel evaluated or judged (46). In addition, feedback based on bodily sensations and represented in the experience in a subtle way is recommended (47), contextualized with the experience and in a non-distracting way (46). This could allow to adapt the therapeutic activity to the patients' current mood and emotions, in a similar way as therapists would adapt traditional interventions to the group's needs. Moreover, this intelligent adaptation would facilitate the definition of the technological intervention of a specific patient: the system could provide different therapeutic activities from which the therapist could choose the most appropriate ones, and the system will be responsible for adapting its content to the specific requirements of that user.

### 3.3 Shaping a modular and collaborative metaverse

**Figure 2:**
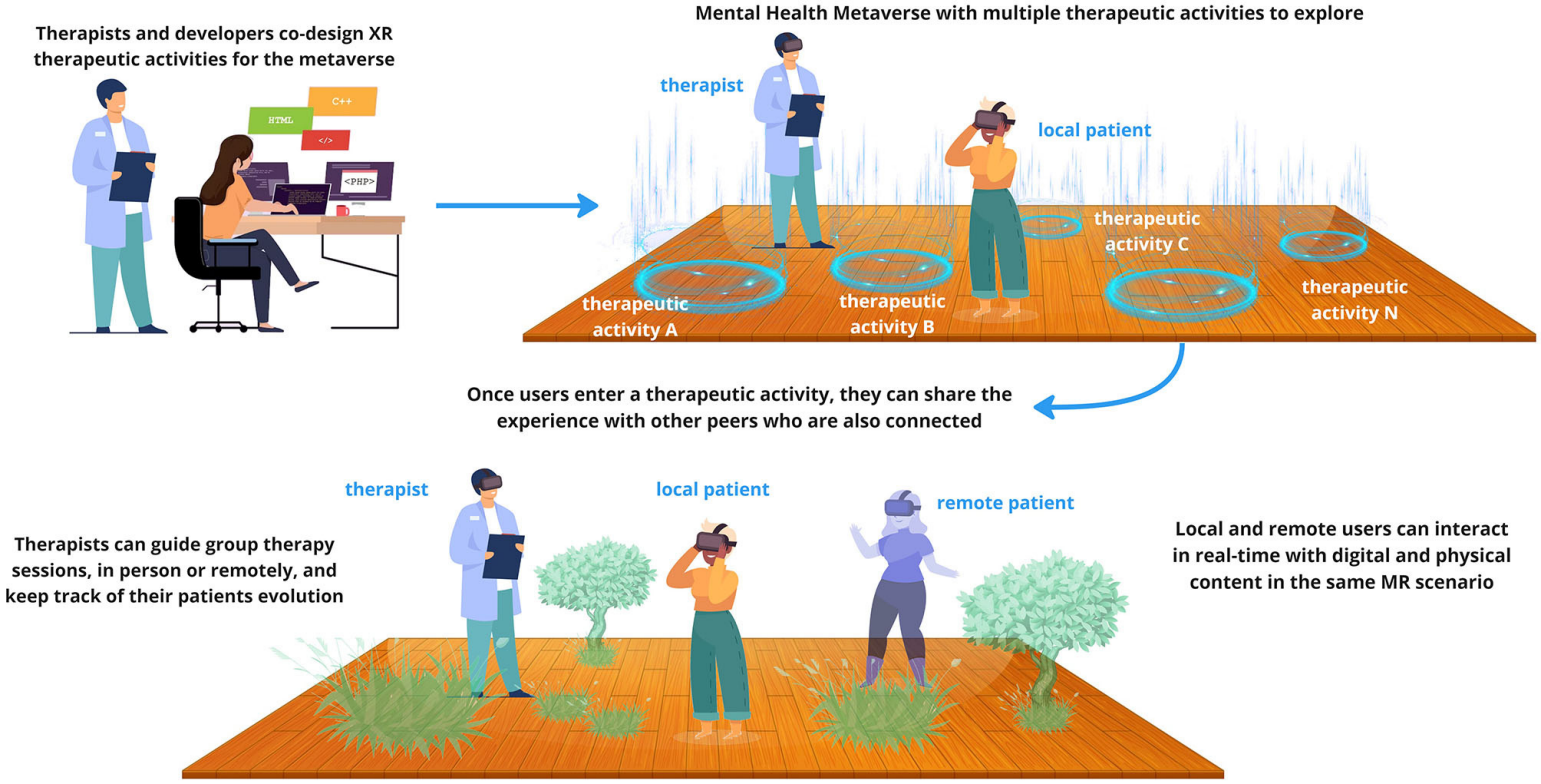
Depiction of the mental health metaverse, showing co-located in-person and remote interactions in Mixed Reality (imaged created with designs from Vecteezy.com, FreePik, and pch.vector from https://www.freepik.com/).

In the emerging mental health metaverse, conventional group therapies can be re-imagined and conducted in dynamic and innovative ways (see Figure 2). Within this interconnected digital realm, participants can come together in their own digitally augmented safe physical space. Users could be offered the opportunity to explore different virtual worlds, each of them with its own particular aesthetic and narrative traits. These worlds would be populated with interactive activities, thoughtfully co-designed by therapists and XR professionals to address specific mental health skills that transcend various pathologies and disorders. For example, one such world could focus on ludic therapeutic activities to avoid rumination and intrusive thoughts, while another world could be aimed to practice self-compassion, providing diverse therapeutic activities where users learn how to deliver kindness toward themselves.

Therapists and mental health professionals would play a vital role in creating, curating and updating these therapeutic activities, incorporating evidence-based approaches and different therapeutic modalities. Working together with XR designers and developers they would shape this collective and adaptive space. The mental health metaverse would act as a complementary and accessible extension, in time and space, of traditional therapy, enabling individuals to reinforce their learning and coping skills beyond scheduled sessions.

The therapeutic activities could be reused in different worlds within the metaverse, to be used by both their patients as well as to people who might be in need, hence reaching other communities and diverse populations. Users have the freedom to access these tailored experiences at their convenience, fostering a self-directed and flexible approach to mental health care. In this way, users would be able to explore different emotional regulation strategies that resonate with them, either accompanied by their therapists or in a self-guided journey, but always in a controlled and supportive social environment.

In these collaborative metaverse, the concept of co-presence plays a pivotal role in shaping the perception of social interactions with other participants. Through the seamless blending of digital and physical-world elements, users can experience a heightened sense of being physically present with others, despite the physical separation. This sense of co-presence can lead individuals to believe that the other participants within the metaverse are real, due to the compelling and realistic nature of the shared virtual environment (48). The psychological connection fostered by this perceived presence of others contributes to a more authentic and emotionally engaging social experience within the metaverse, further enhancing the sense of social realism and immersion in these interactive XR environments.

Additionally, the multi-user nature of metaverse design encourages spontaneous collaboration among users, fostering a supportive and interconnected community of individuals striving for mental wellbeing. On one hand, users would be able to meet other peers in the specific therapeutic activities they are navigating, by collaborating with them within the activities toward a common goal, in a virtual group therapy session, etc. On the other hand, the metaverse offers ways to explore, navigate and discover the different therapeutic activities that are available. In this journey, users can see the avatars of other peers also exploring the area, where spontaneous interactions and conversations might happen. In these way, users can share experiences, provide mutual encouragement, and gain insights from each other's journeys, creating a powerful network of collective healing. Besides the therapeutic activities, therapists could also facilitate group sessions, providing real-time guidance and interventions, while participants can engage in open discussions, emotional expression, and active support for one another.

Through the use of avatars, individuals will be able to represent themselves, interact with their therapist and with other users, navigate and engage with therapeutic content and exercises. The use of avatars is intended to promoting anonymity, reducing the stigma often associated with traditional face-to-face group therapy, and increasing comfort and willingness to engage (33), without diminishing the feeling of co-presence.

In the mental health metaverse, participants can seamlessly join group sessions either remotely or in person, thanks to the versatility of MR technology. This integration enhances accessibility and fosters a diverse and inclusive therapeutic environment. Those who are physically present in the same location can engage with augmented digital elements together, promoting real-time interpersonal interactions and mutual support. Simultaneously, individuals from remote locations can connect to the same virtual space, ensuring they can actively participate and collaborate in the group sessions from the comfort of their own surroundings. This blended approach holds the potential to enhance the sense of connectedness, support, and interpersonal interaction, making therapy sessions more immersive and impactful, enabling collective healing and growth transcending geographical boundaries.

## 4 Conclusion

The metaverse harbors the potential to revolutionize mental health care, breaking down geographical barriers, reducing stigma, and providing accessible and engaging interventions. With its user-driven nature and collaborative environment, the metaverse offers a transformative landscape where collective healing and growth can flourish, empowering individuals on their journey to better mental wellbeing. For mental health practitioners, the metaverse is poised to emerge as an invaluable instrument, as it will enable them to elevate their therapeutic sessions to a higher level, expanding their scope of practice (geographically and socially) and creating ludic environments that enhance accessibility and adherence of treatments.

Anticipating the future, these XR experiences may incorporate haptics, smell, and seamless biofeedback, thereby increasing presence, which could improve the effects of the treatments for the users (49). The rise of generative AI has the potential to significantly enhance the metaverse by improving its intelligent capabilities, facilitating adaptation and personalization. The user experience of MR technologies is expected to greatly improve in the upcoming years, thanks to the advances in hardware devices capabilities and their seamless integration into everyday activities. Efforts to improve accessibility in MR will lead to interfaces designed to accommodate a diverse range of users, including those with physical and cognitive disabilities. This focus on accessibility will make MR experiences more inclusive and usable for a broader audience. While challenges and ethical considerations remain, the mental health metaverse could provided a motivating, social and specialized platform that helps fight the stigma and eases the way to people who currently struggle to find and adhere to their therapeutic process.

## Data availability statement

The original contributions presented in the study are included in the article/supplementary material, further inquiries can be directed to the corresponding author.

## Author contributions

SN-M: Writing—original draft, Writing—review & editing. JS-D: Writing—original draft, Writing—review & editing. PP: Writing—original draft, Writing—review & editing.
